# Myocardial fat quantification using 2D Dixon MRI: feasibility study

**DOI:** 10.1186/1532-429X-11-S1-P107

**Published:** 2009-01-28

**Authors:** Chia-Ying Liu, Alban Redheuil, Ronald Ouwerkerk, Joao Lima, David Bluemke

**Affiliations:** 1 The Johns Hopkins Hospital, Baltimore, MD USA; 2 National Institutes of Health, Bethesda, MD USA

**Keywords:** Intracellular Triglyceride, Myocardial Triglyceride, Gated Press, Unsuppressed Spectrum, Decomposed Water

## Introduction

The concept of fat contained within the myocardium, has recently received attention because of its potential role in diabetic myocardial disease, obesity, and human immunodeficiency virus (HIV) infected individuals [1]. Measurements of myocardial triglycerides in humans have been accessed using proton MR spectroscopy (1H MRS) [2]. 1H MRS provides a precise and reproducible tool for in-vivo quantification of intracellular triglycerides within the sarcolemma. However, the spatial distribution of the fat deposition cannot be accessed by 1H MRS due to its single voxel characteristics. We studied whether the dual-echo Dixon MRI could quantify the fatty content of the myocardium. The fraction of fat was also quantified directly with 1H MRS as an independent method.

## Methods

All MRI/MRS studies were performed using a 3.0 T MR scanner (TrioTim Imager, Siemens) on seven healthy individuals. Myocardial 1H MRS was obtained with a 6-ml voxel positioned in the interventricular septum. Four chamber and short-axis images were acquired by using a breath-hold dual-echo spoiled gradient-recalled echo sequence with TR/TE (In, Out) = 6.3/2.46, 3.69 ms, flip angle = 15° in late diastole. Fat (F) and water (W) images were reconstructed using Matlab. The short-axis water image was used as a reference to contour the epi- and endo-myocardial borders using Mass, and epi-cardial fat was carefully excluded. Contours were transposed to the fat fraction images (defined as F/(F+W) [3]) to calculate the fat fraction of different sectors of the myocardium. MRS was performed with water suppressed ECG gated PRESS, TR/TE = 1 R-R/30 ms, with navigator across the liver-lung interface to reduce of breathing effects. Fat content was quantified with Amares/MRUI and related to water in unsuppressed spectra.

## Results

**Figure 1:**
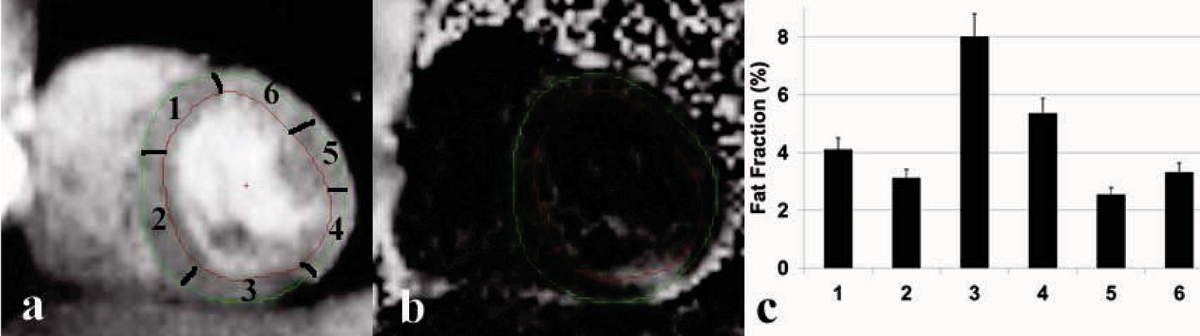
**(a) Decomposed water and (b) Fat fraction images (c) Fat fraction from six segments (contoured on the water image)**. Fat deposition was observed on the inferior lateral wall (segment 3).

**Figure 2:**
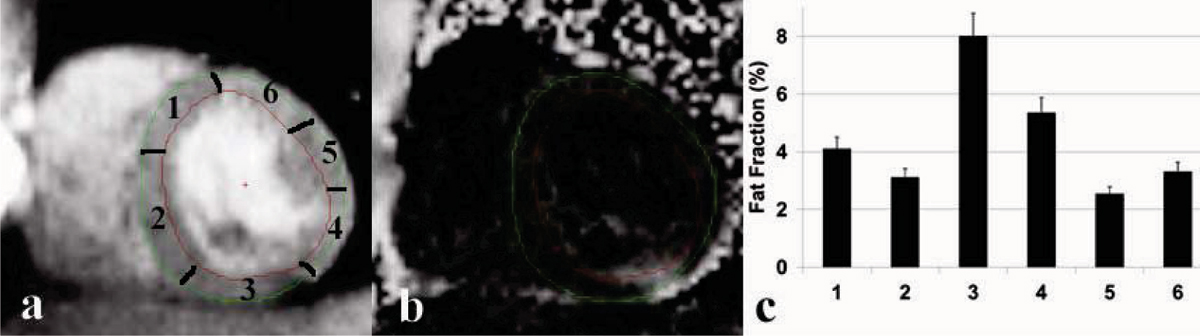
**The fat fraction comparisons shown on the MRI is greater than those of MRS, likey due to noise bias contributing to the fat fraction calculation**.

Figure 1(a) and 1(b) demonstrate decomposed water and fat fraction images from a healthy participant. The fat fractions from six sectors of the myocardium are shown in Figure 1(c). Note the 8% fat deposition on the inferior lateral wall (segment 3). Figure 2 shows the fat fraction based on the in- and out-phase images from the septum (average of section 1 and section 2) of the five volunteers and the comparison to those of 1H MRS.

## Discussion

The dual echo technique showed consistently higher estimates of fat fraction compared to the 1H MRS technique. To accurately quantify the fat fraction, two major issues should be addressed: the effect of tissue relaxation (T_1_ bias) and image noise. T_1_ bias is small however for low fat fraction that we observed. Low signal intensity/image noise may become the dominant source of bias since the magnitude image was reconstructed. Future work will focus on the noise reduction.

## References

[1] Szczepaniak LS (2007). Circ Res.

[2] Meer RW van der (2007). Diabetes.

[3] Liu CY (2007). MRM.

